# Characterization and Evolution of Conserved MicroRNA through Duplication Events in Date Palm (*Phoenix dactylifera*)

**DOI:** 10.1371/journal.pone.0071435

**Published:** 2013-08-08

**Authors:** Yong Xiao, Wei Xia, Yaodong Yang, Annaliese S. Mason, Xintao Lei, Zilong Ma

**Affiliations:** 1 Hainan Key Laboratory of Tropical Oil Crops Biology/Coconuts Research Institute, Chinese Academy of Tropical Agricultural Sciences, Wenchang, Hainan, China; 2 School of Agriculture and Food Sciences and Centre for Integrative Legume Research, The University of Queensland, Brisbane, Australia; 3 Institute of Tropical Bioscience and Biotechnology, Chinese Academy of Tropical Agricultural Science, Haikou, Hainan, China; University of Lausanne, Switzerland

## Abstract

MicroRNAs (miRNAs) are important regulators of gene expression at the post-transcriptional level in a wide range of species. Highly conserved miRNAs regulate ancestral transcription factors common to all plants, and control important basic processes such as cell division and meristem function. We selected 21 conserved miRNA families to analyze the distribution and maintenance of miRNAs. Recently, the first genome sequence in Palmaceae was released: date palm (*Phoenix dactylifera*). We conducted a systematic miRNA analysis in date palm, computationally identifying and characterizing the distribution and duplication of conserved miRNAs in this species compared to other published plant genomes. A total of 81 miRNAs belonging to 18 miRNA families were identified in date palm. The majority of miRNAs in date palm and seven other well-studied plant species were located in intergenic regions and located 4 to 5 kb away from the nearest protein-coding genes. Sequence comparison showed that 67% of date palm miRNA members were present in duplicated segments, and that 135 pairs of miRNA-containing segments were duplicated in *Arabidopsis*, tomato, orange, rice, apple, poplar and soybean with a high similarity of non coding sequences between duplicated segments, indicating genomic duplication was a major force for expansion of conserved miRNAs. Duplicated miRNA pairs in date palm showed divergence in pre-miRNA sequence and in number of promoters, implying that these duplicated pairs may have undergone divergent evolution. Comparisons between date palm and the seven other plant species for the gain/loss of miR167 loci in an ancient segment shared between monocots and dicots suggested that these conserved miRNAs were highly influenced by and diverged as a result of genomic duplication events.

## Introduction

MicroRNAs (miRNAs) are important regulators of gene expression at the post-transcriptional level in a wide range of species, including plants, animals, algae, and some unicellular organisms [1], [2]. Functional, mature miRNA sequences are approximately 20–24 nucleotides (nt) long, and are formed from primary miRNA a few hundred nucleotides long transcribed from miRNA genes [2], [3]. In plants, miRNAs control diverse biological processes, including plant growth and development, reproduction and stress responses [4], [5]. Conserved miRNA families in plants regulate ancestral transcription factors that specify basic meristem functions, organ polarity and separation, cell division, and hormonal control [6], [7]. Hence, the maintenance of these conserved miRNAs is crucial for plants.

Computational approaches to identify conserved miRNAs have been successful in many species. An approach for miRNA prediction using available genome sequence developed in castor bean (*Ricinus communis*) successfully detected 86.6% of miRNAs when tested in Arabidopsis [8]. Sunkar and Jagadeeswaran [9] also performed *in silico* identification of miRNAs in 155 diverse plant species and found 20 conserved miRNA families existing in more than ten plant species. Based on the miRNAs known to date in plants, Cuperus et al. [10] predicted that there are 21 miRNA families conserved between monocots and dicots. Hence, these 21 conserved miRNA families were expected to be found using computational methods in a monocot plant like date palm (*Phoenix dactylifera*).

Date palm is an economically important woody crop grown in tropical and subtropical regions, and is the first species in Palmaceae with released genome sequence [11]. However, the distribution and expansion of miRNA in date palm has not previously been analyzed. The analysis of miRNA in this species will provide the first information of this type for monocot tree species, assisting in evolutionary understanding of miRNAs within this group. Comparison of miRNA between related plant species has previously indicated that extensive chromosomal rearrangements after duplication of miRNA genes play a role in the origin and evolution of miRNAs [12], [13]. However, no conserved block has been identified so far between species from different families. Comparisons of conserved miRNA between plant species could provide clues as to how miRNAs evolve and change over time.

In this study, we used an established analysis pipeline [8] to computationally identify conserved miRNAs in date palm for the first time, and to characterize the distribution and duplication of these conserved miRNAs. Subsequently, we analyzed the evolutionary relationship between conserved miRNAs in date palm and seven other well-studied plant species, determining effects on maintenance and expansion of miRNAs in the different genomes.

## Materials and Methods

### Computational Prediction of Conserved miRNAs in Date Palm and Seven Other Plant Genomes

Mature and hairpin miRNA sequences of the 21 most conserved miRNA families (miR156/157, miR159/319, miR170/171, miR396, miR166, miR160, miR167, miR172, miR169, miR164, miR398, miR399, miR408, miR162, miR168, miR395, miR390, miR397, miR394, miR393, miR482), which exist in more than nineteen land plants in the miRBase, were obtained (miRBase database, Release 19, http://www.mirbase.org, [14]). Using the BLAST algorithm “blastn 2.2.28” with a sensitive BLAST parameter setting (low complexity was chosen as the sequence filter; output file was set as tabular; the default word-match size between the query and database sequences was 7), *Phoenix* genome sequences (http://qatar-weill.cornell.edu/research/datepalmGenome/download.html) were aligned to the downloaded plant miRNA sequences. As miRNA family classifications were based on variation in mature sequences, miRNA sequences in date palm with at least 18 matching nucleotides to mature sequences were selected as candidates. We retrieved the flanking sequences (300 nt upstream and 300 nt downstream; when upstream and/or downstream sequences were shorter than 300 nt, the whole length of sequence was selected) around the matching loci and applied the sequences to the RNAfold program [15] for analyzing secondary structure. Sequences that had folding energy no greater than −18 kcal/mol were chosen for MiRcheck [16] analysis. Unique sequences were detected according to the following parameters [17]: ≤4 mismatches, ≤2 bulged or asymmetrically unpaired nucleotides and ≤2 continuous mismatches in the seed regions, and were retained for further analysis. Genomic sequences for thale cress (*Arabidopsis thaliana* - *ath*), rice (*Oryza sativa* - *osa*), tomato (*Solanum lycopersicum* - *sly*), poplar (*Populus trichocarpa* - *ptc*), soybean (*Glycine max* - *gma*), apple (*Malus domestica* - *mdm)* and orange (*Citrus sinensis* - *csi*) were downloaded from Phytozome version 8.0 (http://www.phytozome.net), and miRNAs in the 21 families were predicted as described above. The predicted miRNAs were then compared with known miRNAs in miRBase for the seven plant species, the majority of miRNAs in miRBase were re-identified in our predictions (Table S1). We reconstructed the history of miRNA genes predicted above using the maximum likelihood procedure implanted in COUNT software [18] with default settings according to Meunier et al. 2013 [19].

### Computational Predictions of Conserved miRNA Targets in Date Palm

All predicted date palm mRNA sequences (28,889 protein coding genes) were downloaded from the website (http://qatar-weill.cornell.edu/research/datepalmGenome/download.html) and 37,080 cDNA sequences isolated from date palm mesocarp were obtained from http://www.biomemb.cnrs.fr/contigs.html [20]. The two sets of date palm mRNA sequences were used to search for potential miRNA targets for the mature miRNA sequences predicted from the date palm genome using Targetfinder1.6 [21], [22]. Briefly, a FASTA search was used to identify the most complementary regions between the mature miRNA query sequences and the date palm mRNA sequences. Alignments from FASTA were turned into RNA duplexes and assessed for mismatches, bulges, gaps (+1 per position) and G:U pairs (+0.5 per position). Penalty scores were doubled at positions 2–13 relative to the 5′ end of the small RNA query sequence. Duplexes were rejected if they had more than one single-nucleotide bulge or gap; if they had more than seven total mismatches, G:U base pairs, bulges and gaps; or if they had more than four total mismatches or four total G:U base pairs.

### Identification of Duplicated miRNAs and Characterization of their Promoters

Using the BLAST algorithm, pairwise alignments between date palm contigs containing miRNAs from the same families were conducted to find paralogous contigs. Redundant sequences with full length matches and sequence identities higher than 98% were excluded. Similarities between duplicated pre-miRNA sequences were analyzed by Blast2. Promoters (TATA box) and enhancers of miRNA genes were predicted from regions 1 kb upstream of pre-miRNAs by using the software TSSP (http://linux1.softberry.com/berry.phtml).

In order to assess miRNA duplication, the genomic locations of the miRNA genes were determined by aligning to the downloaded genome sequences. We retrieved the flanking sequences (10 kb upstream and 10 kb downstream) of miRNA genes and conducted pairwise alignments between sequences containing miRNAs from the same miRNA family. If the flanking sequence had more than 2 kb of matched sequence, the sequence and pre-miRNA were further analyzed by Blast2.

### Identification of Homologous Segments Containing miRNA Genes between the Date Palm Genome and Seven Other Plant Genomes

To identify orthologous segments containing conserved miRNAs between date palm and the seven other plant species, BLASTP was conducted to align protein coding genes between date palm and seven other species, with a cutoff of 1e-10. The BLASTP results were visualized using GenomePixelizer [23]. If there were more than five collinear protein coding gene pairs, the regions were considered to be orthologous. When different segments in one plant genome were homologous to the same date palm contig, the protein coding genes in these segments were aligned with a cutoff of 1e-10. If these segments contained more than five collinear gene pairs, these segments were considered to be paralogous segments in the genome.

## Results

### Distribution of Conserved miRNA Families in the Date Palm Genome and Seven Other Plant Genomes

**Figure 1:**
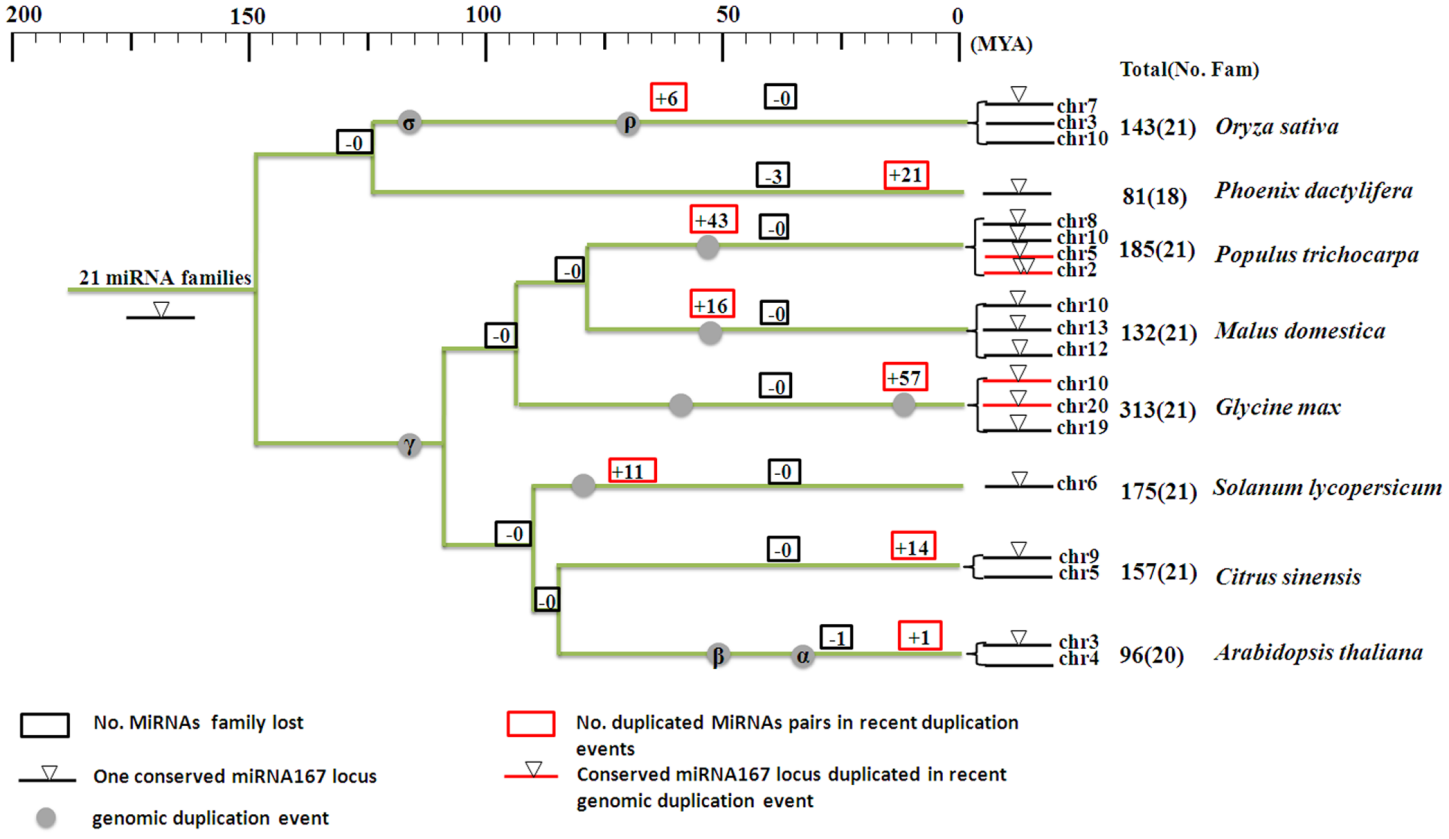
The gain and loss of 21 conserved miRNA families across date palm and seven other plant species. The taxonomic tree was constructed in the Interactive Tree Of Life (http://itol.embl.de/) from taxonomy numbers in NCBI (http://www.ncbi.nlm.nih.gov/Taxonomy/). The history of miRNA genes was reconstructed using the maximum likelihood procedure implanted in COUNT software. The dates of genomic duplication events are from references. The black bars represent the conserved segment, and the triangles indicate the presence of an miR167 locus in the extant plant genomes. Total refers to the total number of miRNA genes in a specific plant, and the number in brackets is the number of miRNA families.

Plant miRNA families which are found in remotely related species are considered to be highly conserved. We selected 21 miRNA families which exist in more than nineteen land plants in the miRBase (ver19) to analyze the distribution and evolutionary maintenance of miRNAs. In total, 53 plant species in 23 families contained miRNAs identified as belonging to the 21 conserved miRNA families (Table S2). Fifteen out of 21 miRNA families also existed in more than 14 families of plant species. Based on the 21 most conserved miRNA families, and using the currently available genome sequence of date palm (*Phoenix dactylifera*), 81 miRNAs were predicted in the date genome (Table 1). As shown in Table 1, the most abundant miRNA family was miR156/157 (12 loci), which has been identified in 45 plant species and which had an average copy number about 18 in the seven other well-studied plant genomes analyzed (Table S2). miR396, miR166, miR172, miR169 and miR395 were also present at multiple loci in date palm, and these miRNAs had the highest average copy number in the other plant species. Noticeably, miR162, miR394 and miR408, which showed low copy number in the other plant species, were not detected in date palm in our analysis. Of 21 miRNA families across the seven plant species, only family - miR482 in Arabidopsis was not detected (Figure 1 and Table S2).

**Table 1:** Predicted miRNAs in date palm (*Phoenix dactylifera*).

miRNA family	No. miRNA	contig(coding genes“–”)^a^		contig(coding genes“+”)^b^		target genes	
		No. miRNA	Flanking(kb)^c^	No. miRNA	Distance(kb)^d^		
miR156	12	5	47.0	3	3.0		Squamosa-promoter binding protein (SPB)-like proteins
miR159	5	1	12.9	1	12.8		myb family transcription factor
miR171	3	1	19.0	2	7.1		scarecrow-like protein 6; nucleosome assembly protein 1;2
miR396	6	2	41.6	2	7.1		growth-regulating factor
miR166	6	3	54.2	1	6.2		homeobox-leucine zipper protein
miR160	3	3	33.0	0	unknown		auxin response factor
miR167	3	1	54.6	2	9.5		auxin response factor; serine threonine protein
miR172	6	4	28.5	2	8.1		Floral homeotic protein; eIf4g (eukaryotic translation initiation factor4g)
miR169	7	4	27.3	2	7.4		nad kinase 1
miR164	6	3	13.3	1	7.9		NAC domain containing protein
miR398	2	1	46.6	0	unknown		unknown
miR399	3	1	16.1	1	3.1		AAA-type ATPase family protein; l-asparaginase l-asparagine amidohydrolase
miR168	5	5	54.1	0	unknown		argonaute protein; ATP-dependent RNA helicase
miR395	7	1	21.4	2	4.2		malate dehydrogenase; ATP sulfurylase
miR390	1	–	–	–	–		Protein MLO; protein defective in meristem silencing 3
miR397	2	1	29.4	1	3.3		chromatin remodeling complex subunit
miR393	3	1	43.6	2	4.3		auxin signaling F-box 3 protein;
miR482	1	–	–	–	–		unknown
Total	81	37	33.9	22	6.5		

a “–” Date palm contig (≥6.5kb) has no protein-coding genes;

b “+” Presents of protein-coding genes in the contig;

c Length of miRNAs flanking region without protein-coding genes;

d Distance between miRNA and the nearest protein-coding gene/s.

**Figure 2:**
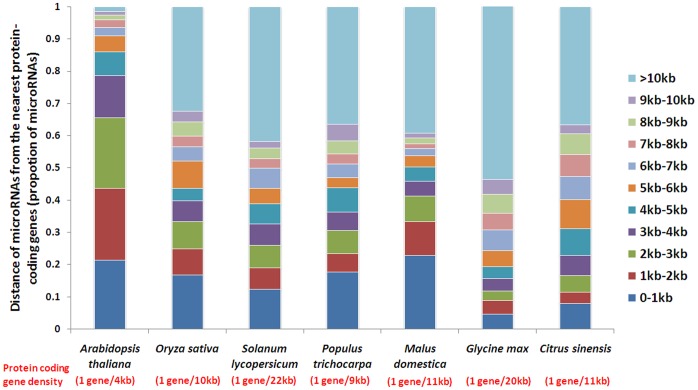
Distance of miRNAs from nearest protein coding genes in seven model plants. For distances ranging from 0 to 1 kb, miRNAs were included to be inside genic regions. The protein coding gene densities were calculated from the following data: *Arabidopsis thalian*a (27228 genes/115.4 Mb), *Oryza sativa* (40577 genes/398 Mb), *Solanum lycopersicum* (34721 genes/760 Mb), *Populus trichocarpa* (45654 genes/410 Mb), *Malus domestica* (57386 genes/603.9 Mb), *Glycine max* (46430 genes/950 Mb), *Citrus sinensis* (29445 genes/320.5 Mb).

MiRNA flanking sequences were analyzed to characterize the genomic location of the miRNAs. The majority of date palm pre-miRNAs (78 loci) predicted in the date palm genome were located in the intergenic regions and not related to transposable elements. One miRNA gene was located in a known genic region, while two other miRNA genes overlapped with predicted genic regions with unknown function. The average gene density in the draft genome of date palm is approximately one protein coding gene per 13 kb (28889 genes/380 Mb). Analysis of miRNA flanking sequences showed that 22 miRNAs were close to protein coding genes, with an average distance of about 6.5 kb to the proximate protein coding genes, and 37 miRNA genes (contig length ≥6.5 kb) had no flanking protein coding genes within an average of 33 kb (Table 1). Further analysis showed that seven other plant species also had a similar distribution of miRNA proximities to protein coding genes (Figure 2: all but *Arabidopsis thaliana*) had about 50% of their miRNA genes located 4 kb –5 kb away from protein coding genes despite divergent gene densities between different plants. The proportion of miRNAs located more than 10 kb away from a protein coding genes, ranged from 1% (*Arabidopsis thaliana*) to 54% (*Glycine max*), *Solanum lycopersicum* and *Glycine max*, which have gene densities of one gene per 22 kb and one gene per 20 kb, respectively, had the higher proportion of miRNAs located more than 10 kb away from protein coding genes than the other plant species.

MiRNA target prediction conducted using Targetfinder showed that fourteen miRNA families had predicted target genes in both sets of mRNA sequence (from predicted gene models and from cDNA sequences in date palm mesocarp). However, target genes for miR167 were detected only in predicted gene models, and miR171 had predicted target genes only in mesocarp cDNA sequences. The prediction of target genes for miRNA families showed that miRNAs in the same family tended to target the same gene family (Table 1 and Table S3, the frequency of target genes belonging to same gene family versus the frequency of target genes belonging to different gene families, Student’s t-test, P<0.001). However, miRNA156, miRNA159 and miR172 targeted more than one gene family. Targeted gene families were mostly involved in developmental processes and auxin response factors were targeted by two miRNA families - miR160 and miR167.

### MiRNA Expansion through Duplication in the Date Palm Genome

**Figure 3:**
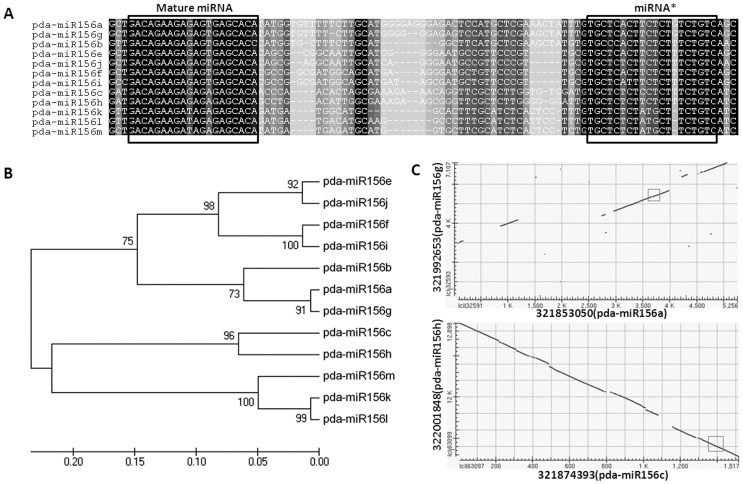
Alignment of multiple sequences and phylogenic analysis of microRNA156 pre-miRNAs in date palm. A) Alignment of twelve pre-miRNA sequences of miR156. The box to the right indicates the mature miRNA region. The box to the left indicates the star miRNA region. B) Phylogenic tree (formed by Neighbor Joining) for miRNA in the miR156 family. C) Blast2 results for two pairs of paralogous date palm contigs containing pre-miR156. Squares highlight the location of the pre-miRNAs.

 As increasing gene copies tend to protect against loss of the gene in the genome, miRNA duplication could assist in maintenance of miRNAs. Of the 18 miRNA families identified in date palm, 16 (89%) contained more than one pre-miRNA (Table 1). Since the loop regions of pre-miRNA are highly variable, miRNAs with highly similar pre-miRNAs may originate from duplication. Sequence alignment of pre-miRNAs within families showed that many miRNAs were highly similar, with differences of only several nucleotides. Within the miR156 family, sequence alignments showed that miR156e/j, miR156f/i, miR156a/g, miR156c/h, and miR156k/l had high similarity, and the 12 miRNAs could be divided into five groups based on multiple sequence alignments (Figure 3A–B and Figure S1). Further sequence comparison between date palm contigs containing miR156 showed that four pairs of miRNAs (miR156e/j, miR156f/i, miR156a/g, and miR156c/h) had highly similar flanking sequences (Table 2). As shown in Figure 3C, although divergence due to insertions/deletions occurred in the flanking sequences of miR156a/g and miR157c/h, the miRNAs themselves were preserved.

**Table 2:** Duplicated miRNA pairs detected in paralogous contigs of date palm.

miRNA family	miRNAs in paralogous contigs	No. Promoters	pre-miRNA sequence similarity
miR156	pda-miR156a/g	2/2	94%
	pda-miR156c/h	1/3	87%
	pda-miR156e/j	1/1	97%
	pda-miR156f/i	1/2	96%
miR159	pda-miR159c/d	−/1	96%
miR396	pda-miR396b/c	2/2	83%
miR166	pda-miR166a/b/f	−/1/1	75%–76%
miR160	pda-miR160a/c	1/3	98%
miR167	pda-miR167a/b	1/2	84%
miR172	pda-miR172a/b	1/3	93%
	pda-miR172c/d	1/1	88%
	pda-miR172e/f	3/3	75%
miR169	pda-miR169a/e	−/1	75%
	pda-miR169b/f	1/1	87%
	pda-miR169c/d	1/2	90%
miR164	pda-miR164a/d	−/1	86%
	pda-miR164b/e	2/2	94%
	pda-miR164c/f	−/−	90%
miR168	pda-miR168b/e	1/1	93%
miR395	pda-miR395(a–b)*/(d–e)/(f–g)/c	−/1/1/−	93%–94%
miR393	pda-miR393a/c	1/−	90%
Total	48	56	

* miRNA in brackets represent tandem miRNAs.

Since pairs of miRNAs located on paralogous contigs were considered to originate from duplication, date palm contigs containing miRNAs were aligned to identify paralogous contigs and to analyze the miRNA duplication events. Table 2 shows the 21 groups of paralogous contigs with the highest matches for non-protein coding regions. Of 16 families containing more than one pre-miRNA, 48 miRNA members in 12 families (79% of miRNA members in 75% of families) were putatively involved in duplication events. In the miR164, miR172 and miR395 families, all miRNA members were involved in duplication events.

 As indicated in Table 2, 19/21 replicated miRNAs (90%) were present in two copies, with two exceptions: miR166 (three copies) and miR395 (four copies). However, these duplicated pre-miRNAs showed a range of sequence similarities from 75–98%, and 12 pairs of duplicated miRNAs (57%) had sequence similarities higher than 90%. Moreover, the conservation of core promoters of these duplicated miRNAs varied between and within miRNA families. Although 55 putative core promoters of these duplicated miRNAs were predicted for the 40 miRNAs which had 1 kb of upstream sequence available, only 10/16 of the duplicated pairs (63%) had the same predicted number of core promoters.

In addition to analysis of miRNA duplications between paralogous contigs, miRNA-containing tandem repeats were also detected in miR395 and miR396. In the miR395 family, three pairs of tandem duplications (MiR395d/e, miR395a/b and miR395f/g) were detected. The distance between pairs of tandem miRNAs ranged from 107 to 115 bp. The sequence similarity between tandemly duplicated miRNAs was about 75%, much lower than the sequence similarity observed for duplication between different contigs, indicating that these tandem duplications happened before contig duplication (Figure S2). In the miR396 family, miR396e/f was a tandem duplication pair with pre-miRNA sequence similarity of about 85%, and a short (135 bp) distance between tandem repeats.

### Genomic Duplication and miRNA Expansion in Date Palm and Seven Other Plant Genomes

As genomic duplication is an important mechanism for boosting gene and sequence copy number, we analyzed the gain and loss of conserved miRNAs across different plants in duplicated genomic regions. Among the seven well studied plants, most species had two or more duplicated miRNA-sequence-containing regions. Sequence alignments of 20 kb sequences containing miRNAs detected a total of 135 duplicated regions in the seven plants genomes, distributed as follows: *Oryza sativa* (6), *Populus trichocarpa* (43), *Malus domestica* (16), *Glycine max* (58), *Solanum lycopersicum* (11), *Citrus sinensis* (14) and *Arabidopsis thaliana* (1) (Table S4 and Figure 1). These duplicated regions included miRNAs from all 21 families, and duplication on family miR156 duplicated was detected in all four species.

These duplicated regions showed high identity in the non-coding regions flanking miRNAs, indicative of recent duplication events. Soybean (*Glycine max*) had the highest copy number of miRNAs detected with 57 pairs of duplicated segments probably arising from a whole genome duplication event [24]. More duplicated miRNAs were observed in plants with more recent whole genome duplication events: apple (*Malus domestica*), poplar (*Populus trichocarpa*) and soybean (*Glycine max*) had high miRNA gene copies and conservation of the 21 conserved miRNA families (Figure 1 and Table S4).

### Conservation of Orthologous Blocks of miRNA in Date Palm and Other Plants

Since the 18 analyzed miRNA families in date palm exist in many plants, date palm pre-miRNAs were compared to those of other species in miRBase. Thirteen date palm pre-miRNAs showed sequence similarities of 82–97% with other plant species pre-miRNAs, with aligned regions of more than 50 bp covering the loop region. These thirteen conserved pre-miRNAs belonged to the miR156 (6), miR159 (4), miR160 (2) and miR170 (1) families. However, the flanking sequences of these matched date palm miRNAs were non-coding regions, and no sequence homology was detected with flanking genome sequences.

To identify whether orthologous blocks were shared between plant species for conserved miRNAs, date palm contigs containing both miRNA and protein-coding genes were compared between the date palm genome and the genomes of seven other land plants. These plants were from seven different families, and had available genome sequences with identified miRNA genes in miRBase. The alignments indicated that four date palm contigs had 36 orthologous segments in the other seven species (Table 3). As shown in Table 3, Contig PDK_30s943301 containing pda-miR167c was the most conserved contig, and was found in all seven plant genomes (18 copies in total). Six out of seven plants had more than one copy of this segment: *Populus trichocarpa* had the highest copy number (4) and *Solanum lycopersicum* had only a single copy (1). Detailed alignment results for protein-coding genes conserved between date palm and *Arabidopsis thaliana*/*Oryza sativa* (Figure S3) showed that a collinear relationship exists between date palm contig PDK_30s943301 and *Arabidopsis thaliana*/*Oryza sativa* chromosomes. Of the three orthologous regions identified, conserved genes in chromosome 7 of *Oryza sativa* were the most dispersed (Figure S3).

**Table 3:** Orthologous segments between date palm (*Phoenix dactylifera*) and seven other plant species.

Contigs name	Species	No. contigs	Location	miRNA
PDK_30s943301	ath^b^	2	chr3(11)^c^,chr4(11)	miR167(1)
(miR167)^a^	csi	2	chr5(16),chr9(18)	miR167(1)
	gma	3	chr10(7),chr19(8),chr20(6)	miR167(3)
	mdm	3	chr11(10),chr13(15),chr16(7)	miR167(3)
	osa	3	chr3(9),chr7(13),chr10(6)	miR167(1)
	ptc	4	chr2(11),chr5(12),chr8(16),chr10(13)	miR167(6)
	sly	1	chr9(17)	miR167(1)
		**18**		
PDK_30s6550926	csi	2	chr6(13),chr7(10)	miR395(0)
(miR395)	gma	1	chr6(5)	miR395(0)
	mdm	3	chr9(6),chr13(7),chr17(11)	miR395(0)
	osa	3	chr2(8),chr8(9),chr12(6)	miR395(0)
	ptc	1	chr17(10)	miR395(0)
		**10**		
PDK_30s740551	csi	1	chr5(9)	miR171(0)
(miR171)	gma	2	chr4(5),chr6(6)	miR171(0)
	mdm	1	chr6(7)	miR171(0)
	ptc	2	chr1(6),chr3(5)	miR171(0)
	sly	1	chr3(6)	miR171(0)
		**7**		
PDK_30s732171	csi	1	chr6(12)	miR393(0)
(miR393)				
Total	7	36		16

a MicroRNA located in the contig;

b Arabidopsis thaliana (ath), Citrus sinensis (csi), Glycine max (gma), Oryza sativa (osa), Solanum lycopersicum (sly), Populus trichocarpa (ptc), and Malus domestica (mdm).

c Numbers in brackets represent numbers of conserved genes in orthologous blocks.

Among the four date palm contigs, contig PDK_30s943301 had the highest number of orthologous segments, existing in seven plants as well as in date palm contigs. Moreover, thirteen orthologous segments of PDK_30s943301 had more than ten conserved genes, while more than 50% of genes in PDK_30s943301 were conserved in some regions of remotely related plant species *Citrus sinensis* (19), *Populus trichocarpa* (17) and *Solanum lycopersicum* (18). Scaffold PDK_30s943301 was an ancient conserved block between monocots and dicots, and the date of divergence between these groups could not be assessed as the Ks value was saturated (>2). Contig PDK_30s6550926, which contained a tandem duplication of miR395, was found in orthologous segments in six plants, with a total of 13 copies. However, no miR395 members existed in orthologous regions of the five plants. Contig PDK_30s740551 was detected in eight orthologous segments in five species and contig PDK_30s732171 was detected one orthologous segment in *Citrus sinensis*, but no corresponding miRNA families were found in these regions.

### Evolution of an miR167 Locus within a Conserved Contig between Plant Species

**Figure 4:**
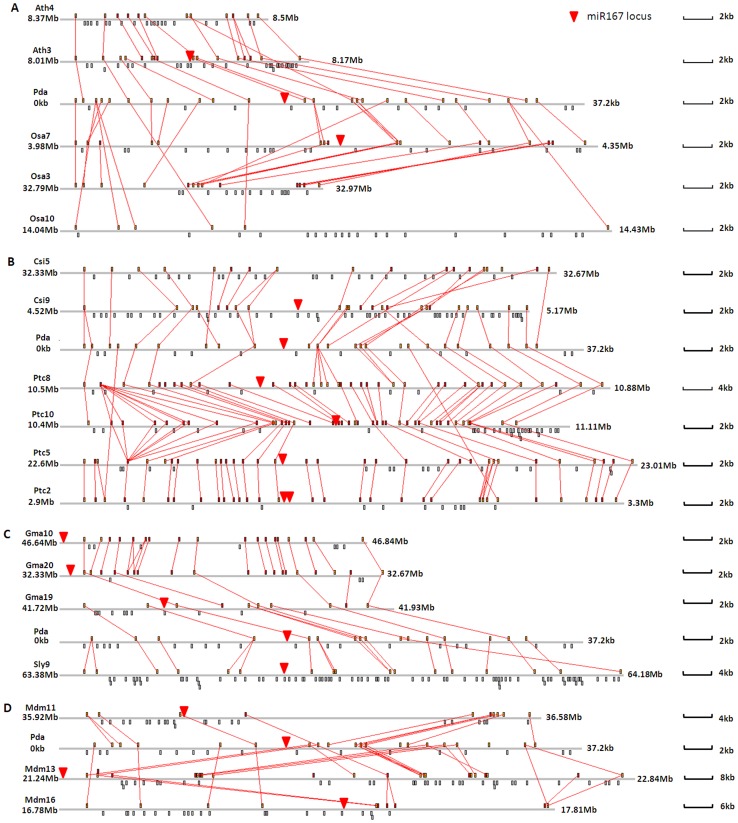
Relationship between date palm contigPDK_30s943301 containing an miR167 locus and orthologous segments from seven other plant species. Each panel shows conserved genes aligned by red lines. Red arrows represent the miR167 loci. The grey horizontal lines represent the homologous regions from different chromosomes. The dots above the grey horizontal lines indicate conserved genes that could be aligned between species, while dots below the grey horizontal lines indicate genes that could not be aligned between species. Ath3 is short for chromosome 3 of *Arabidopsis thaliana*. Pda, osa, csi, ptc, gma, sly and mdm are short for *Phoenix dactylifera*, *Oryza sativa*, *Citrus sinensis*, *Populus trichocarpa*, *Glycine max*, *Solanum lycopersicum*, and *Malus domestica*, respectively.

Detailed alignments and comparison of orthologous regions in date palm contigPDK_30s943301 (containing one miR167 locus) were conducted to highlight the variation and divergence between date palm and the other reference genomes (Figure 4). Of 18 orthologous regions, 14 (78%) had an miR167 locus in the collinear region, and four orthologous regions - Ath4, Osa3, Osa10 and Csi5 - lost the miR167 locus. Although miR167 was conserved in most orthologous regions from different plants, the flanking genes varied. In Osa7, the miR167 locus was shuffled to a nearby region. In Ptc2, the miR167 locus was tandemly duplicated. The distance between flanking genes and the miRNAs differed in all orthologous regions. Moreover, the majority of orthologous regions from tree species *Populus trichocarpa*, *Malus domestica* and *Citrus sinensis* had larger, expanded orthologous regions relative to herbaceous species *Arabidopsis thaliana*, *Oryza sativa* and *Glycine max*.

miR167 was predicted to be involved in auxin response transcription factors, which are important for plant architecture. Our analysis of orthologous contigs containing miR167 between remotely related plant species indicated that genomic duplications significantly influenced the conservation and expansion of miR167 locus (Figure 1). Plants belonging to the Fabids (*Populus trichocarpa*, *Malus domestica* and *Glycine max*) had the highest number of conserved segments, and all miR167 loci were preserved. However, only one of two orthologous segments in *Arabidopsis thaliana, Oryza sativa* and *Citrus sinensis* had an miR167 locus. *Phoenix dactylifera* and *Solanum lycopersicum* had one orthologous miR167 locus, indicative of the ancient state of this unduplicated region.

## Discussion

In this study, we predicted and characterized conserved miRNAs in date palm for the first time, and compared miRNA distribution and presence in orthologous regions between date palm and seven other well-studied plants. The majority of miRNAs were located in intergenic regions 4 to 5 kb away from the nearest protein-coding genes, and we determined that duplication events are the major driving force behind the evolution of miRNAs. Duplicated miRNA pairs showed different levels of divergence in the pre-miRNA and promoter regions, and whole genome duplication events could greatly boost miRNAs copy numbers. Further analysis of orthologous blocks between date palm and plant species from other families indicated that most contigs had no collinear relationship, and contigs conserved between species were divergent in their maintenance of miRNA loci. These observations suggest that genomic duplication is a major force for the maintenance of these conserved miRNAs, and is responsible for the different tendencies to accumulate miRNAs between species.

Conserved miRNA families could be predicted computationally due to the high level of conservation and widespread presence of these miRNAs in plants [9], [25]. Sunkar (2008) found ∼21 miRNA families to be conserved between dicots and monocots using *in silico* identification. In date palm, computational prediction of miRNAs from these conserved miRNA families revealed similar numbers of miRNA members compared to other well-studied plant species (Table 1). The majority of the conserved miRNAs in date palm were located in the intergenic regions, and a high proportion of these old miRNA families in date palm and in the seven other plants were located more than 4 to 5 kb away from protein coding genes (Figure 2). The predicted target genes of the miRNAs in date palm were consistent with target genes previously identified in *Arabidopsis* and rice [16], [26], [27]. Hence, the detection method we used to predict conserved miRNAs seems to be efficient for novel genome sequences.

Conserved miRNAs between remotely related plants, or ‘old’ miRNAs, were greatly influenced by genomic duplication events, which many species have undergone [24], [28], [29], [30]. Analysis of miRNA family members showed that duplication events contribute to the replication and maintenance of miRNAs in date palm (Table 2). Moreover, 135 duplicated miRNAs were detected in other species: *Arabidopsis thaliana*, *Citrus sinensis*, *Solanum lycopersicum*, *Oryza sativa*, *Populus trichocarpa*, *Malus domestica* and *Glycine max*. High sequence identities between duplicated segments in the flanking non protein-coding sequences suggest that these miRNAs were widely influenced by recent whole genome duplication events. Soybean had the highest miRNA copy number detected with 57 pairs of duplicated segments probably arising from recent whole genomic duplication. Apple, poplar and soybean, all with very recent genome duplication events [24], [30], [31], had the highest detected miRNA copy number and no miRNA family loss. The comparison of protein-coding genes showed that old duplication events could influence the expansion of conserved miRNAs in *Oryza sativa*, *Arabidopsis thaliana and Glycine max* [12], [13], [27], [32], [33]. The tandem duplication of miR395 detected in date palm was consistent with other the tandem duplication events detected in *Arabidopsis*, tomato, rice, *Medicago* and poplar [25], [34], [35]. The frequent observation of paralogous contigs between miRNAs indicated duplications were a dominant force for evolution of conserved miRNAs. These results suggest that genomic duplication exerts an influence on maintenance of old miRNAs in remotely related plants in both the dicots and monocots.

Old miRNAs are conserved between many land plants [9], [36], [37]. In our study, the majority of miRNA-containing contigs shared between date palm and other species were observed to be not well conserved, but to preferentially retain miRNA loci (Table 3). However, one contig containing the miR167 locus showed collinearity between both monocots and dicots (Figure 1). miR167 targets auxin response factors, which are the transcription factors that regulate the expression of auxin-responsive genes and play critical roles in plant development [38], [39], [40]. Previous studies have observed that there are two miR167 loci [12], and our analysis showed that one of the two loci was preserved in genomic duplication. The results suggest that this miR167 locus may be crucial for both monocots and dicots, as it is far more highly preserved in the process of genomic duplication and new species formation than other miRNAs.

The miR167 locus along with its flanking region was duplicated in seven species from different families, with the miRNA loci maintained in this region (Figure 1 and 4). Our analysis indicated that these ancient and conserved segments varied in their maintenance of miR167. This miRNA had one copy in diploid plants which had undergone only ancient genomic duplications, such as rice, *Arabidopsis* and orange, but had three to four copies in polyploid species apple, soybean and poplar, which had relatively recent genome-wide duplication events [9], [30], [41]. Our analysis of duplicated miRNA-containing segments indicated that two miR167 segments in poplar and soybean were duplicated very recently. These observations imply that copies of miR167 can be lost or retained due to different species evolutionary histories, with genome-wide duplication as a major factor, but that conservation of a single copy is universally selected for.

In addition to the maintenance and expansion effects of miRNAs from genomic duplication events, the divergence of duplicated miRNAs was also detected (Table 2). Our analysis of pre-miRNA sequences and promoters indicated that the duplicated miRNAs have undergone noticeable divergence. Among duplicated genomic segments containing miRNAs, the loss of miRNA genes and shift of miRNA locations was also detected (Figure 4). Research into miRNAs which arose from genomic duplication indicated that these miRNAs have undergone dispersal and diversification, similar to the processes that drive the evolution of protein gene families [27]. Thus, miRNA replicated through genome duplication may not only contribute to the conservation of miRNAs but also be a source of new miRNA via sub-functionalization and change in expression pattern.

In short, we evaluated the distribution and expansion of conserved miRNA families in date palm. Adding this monocot species into a comparative analysis between different land plants, we analyzed the evolution of an miR167 locus in an orthologous DNA segment shared between eight species from different families. The gain and loss of the conserved miR167 loci implies that conserved miRNAs are maintained despite sequence divergence between different plants as a result of genomic duplication.

## Supporting Information

Figure S1 **Blast2 alignments for pre-miRNA sequences of pda-miR156a, pda-miR156b and pda-miR156g.**
(PDF)

Figure S2 **Phylogenic analysis of microR395 pre-miRNAs in date palm (** ***Phoenix dactylifera*** **).**
(PDF)

Figure S3 **Conserved protein coding genes between date contigPDK_30s943301 (Accession: 322483308, pda-miR167c located) and** ***Arabidopsis thaliana*** **/** ***Oryza sativa*** **.**
(PDF)

Table S1 **Detailed information for miRNAs predicted in** ***Arabidopsis thaliana*** **(ath),** ***Citrus sinensis*** **(csi),** ***Glycine max*** **(gma),** ***Oryza sativa*** **(osa),** ***Solanum lycopersicum*** **(sly),** ***Populus trichocarpa*** **(ptc), and** ***Malus domestica*** **(mdm).**
(XLS)

Table S2 **The number of individual members in a miRNA family of seven well-studied plants.**
(XLS)

Table S3 **Target genes for the miRNA genes in date palm (** ***Phoenix dactylifera*** **).**
(XLS)

Table S4 **Recently duplicated miRNAs pairs in seven well-studied plants.**
(XLS)
