# Supplementary material for: Characterization and Evolution of Conserved MicroRNA through Duplication Events in Date Palm (Phoenix dactylifera)
Source: PLoS One. 2013 Aug 8;8(8):e71435. doi: 10.1371/journal.pone.0071435 (PMC3738527; doi:10.1371/journal.pone.0071435)
Supplement: Figure S1 — Blast2 alignments for pre-miRNA sequences of pda-miR156a, pda-miR156b and pda-miR156g. (PDF) [file pone.0071435.s001.pdf]

```
pda-miR156a GCTGACAGAAGAGAGTGAGCACATATGGTGTTTTTCTTGCATGGGGAGGGAGACTCCATGCTCGAAACTATTTGTGCTCACTTCTCTGTCTGTCAGC
pda-miR156b GTTGACAGAAGAGAGTGAGCACCCATGGTG-CTTTCTTGCAT---------GGCTTCATGCTCGAAGCTATGTGTGCCCACTTCTCTTTCTGTCAAC

pda-miR156a GCTGACAGAAGAGAGTGAGCACATATGGTGTTTTTCTTGCATGGGGAGGGAGACTCCATGCTCGAAACTATTTGTGCTCACTTCTCTGTCTGTCAGC
pda-miR156g GCTGACAGAAGAGAGTGAGCACATATGGTGTTTT-CTTGCATGGGG--GGAGAGTCCATGCTCGAAGCTATTTGTGCTCACTTCTCTGTCTGTCAGC

pda-miR156g GCTGACAGAAGAGAGTGAGCACATATGGTGTTTTCTTGCATGGGGGGAGAGTCCATGCTCGAAGCTATTTGTGCTCACTTCTCTGTCTGTCAGC
pda-miR156b GTTGACAGAAGAGAGTGAGCACCCATGGTGCTTTCTTGCATGGC-------TTCATGCTCGAAGCTATGTGTGCCCACTTCTCTTTCTGTCAAC
```

Figure S1 Blast2 alignments for pre-miRNA sequences of pda-miRNA156a, pda-miRNA156b and pda-miRNA156g.
