# Supplementary material for: Characterization and Evolution of Conserved MicroRNA through Duplication Events in Date Palm (Phoenix dactylifera)
Source: PLoS One. 2013 Aug 8;8(8):e71435. doi: 10.1371/journal.pone.0071435 (PMC3738527; doi:10.1371/journal.pone.0071435)
Supplement: Figure S2 — Phylogenic analysis of microR395 pre-miRNAs in date palm ( Phoenix dactylifera ). (PDF) [file pone.0071435.s002.pdf]

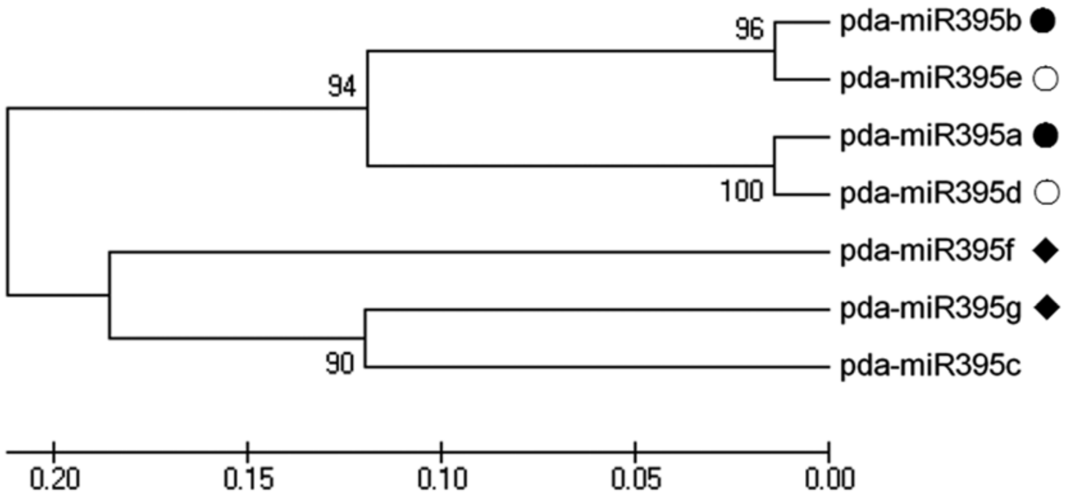

Figure S2. **Phylogenic analysis of microRNA395 pre-miRNAs in date palm (*Phoenix dactylifera*).** Phylogenic tree was formed by Neighbor Joining. The same symbols (●, ○, ◆) on the left of the miRNAs represent pairs miRNAs belong to tandem duplications.
