# Supplementary material for: Characterization and Evolution of Conserved MicroRNA through Duplication Events in Date Palm (Phoenix dactylifera)
Source: PLoS One. 2013 Aug 8;8(8):e71435. doi: 10.1371/journal.pone.0071435 (PMC3738527; doi:10.1371/journal.pone.0071435)
Supplement: Figure S3 — Conserved protein coding genes between date contigPDK_30s943301 (Accession: 322483308, pda-miR167c located) and Arabidopsis thaliana / Oryza sativa . (PDF) [file pone.0071435.s003.pdf]

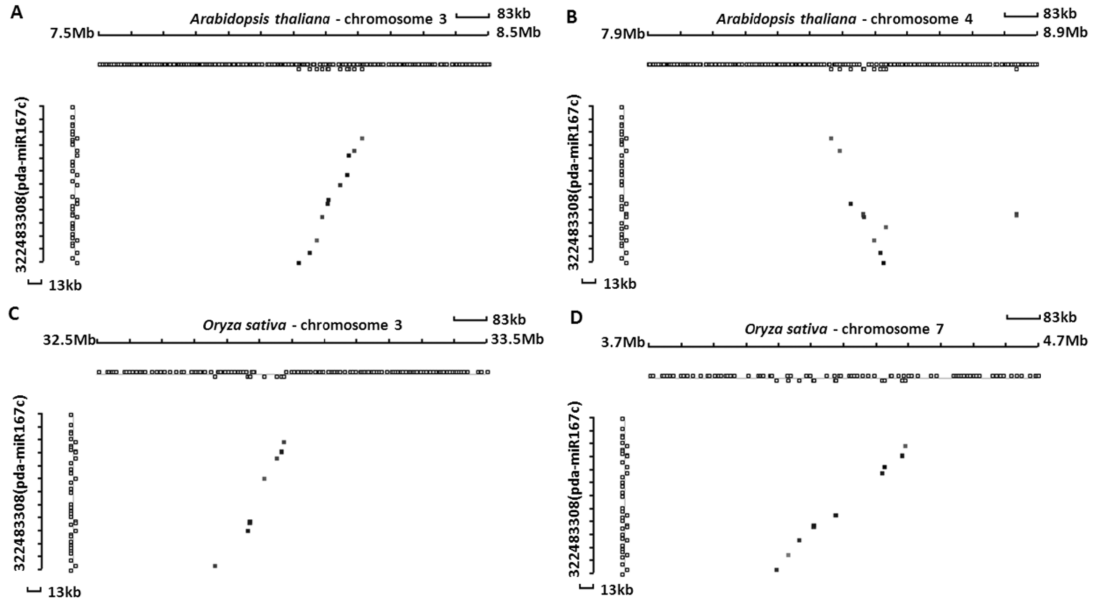

Figure S3 Conserved protein coding genes between date contigPDK_30s943301 (Accession: 322483308, pda-miR167c located) and *Arabidopsis thaliana*/*Oryza sativa*. A - B) Conserved protein coding genes between date palm contigPDK_30s943301 and *Arabidopsis thaliana* chromosomes 3 and 4. C - D) Conserved protein coding genes between date palm contigPDK_30s943301 and *Oryza sativa* chromosomes 3 and 7.
